# Sustained reduction in vaccine-type invasive pneumococcal disease despite waning effects of a catch-up campaign in Kilifi, Kenya: A mathematical model based on pre-vaccination data

**DOI:** 10.1016/j.vaccine.2017.07.019

**Published:** 2017-08-16

**Authors:** John Ojal, Stefan Flasche, Laura L. Hammitt, Donald Akech, Moses C. Kiti, Tatu Kamau, Ifedayo Adetifa, Markku Nurhonen, J. Anthony G. Scott, Kari Auranen

**Affiliations:** a KEMRI-Wellcome Trust Research Programme, Centre for Geographic Medicine-Coast, Kilifi, Kenya; b Department of International Health, Johns Hopkins Bloomberg School of Public Health, Baltimore, MD, USA; c Department of Infectious Disease Epidemiology, Faculty of Epidemiology and Population Health, London School of Hygiene and Tropical Medicine, London, United Kingdom; d Kenya Ministry of Health, Nairobi, Kenya; e Department of Public Health Solutions, National Institute for Health and Welfare (THL), Finland; f Department of Mathematics and Statistics, University of Turku, Finland

**Keywords:** Pneumococcal conjugate vaccine, Nasopharyngeal carriage, Kenya, Mathematical model

## Abstract

**Background:**

In 2011, Kenya introduced the 10-valent pneumococcal conjugate vaccine together with a catch-up campaign for children aged <5 years in Kilifi County. In a post-vaccination surveillance study based in Kilifi, there was a substantial decline in invasive pneumococcal disease (IPD). However, given the continued circulation of the vaccine serotypes it is possible that vaccine-serotype disease may re-emerge once the effects of the catch-up campaign wear off.

**Methods:**

We developed a compartmental, age-structured dynamic model of pneumococcal carriage and invasive disease for three serotype groups: the 10-valent vaccine serotypes and two groups of non-vaccine serotypes based on their susceptibility to mutual competition. The model was calibrated to age- and serotype-specific data on carriage and IPD in the pre-vaccination era and used to predict carriage prevalence and IPD up to ten years post-vaccination in Kilifi. The model was validated against the observed carriage prevalence after vaccine introduction.

**Results:**

The model predicts a sustained reduction in vaccine-type pneumococcal carriage prevalence from 33% to 8% in infants and from 30% to 8% in 1–5 year olds over the 10-year period following vaccine introduction. The incidence of IPD is predicted to decline across all age groups resulting in an overall reduction of 56% in the population, corresponding to 10.4 cases per 100,000 per year. The vaccine-type IPD incidence is estimated to decline by 83% while non-vaccine-type IPD incidence is predicted to increase by 52%. The model's predictions of carriage prevalence agrees well with the observed data in the first five years post-vaccination.

**Conclusion:**

We predict a sustained and substantial decline in IPD through PCV vaccination and that the current regimen is insufficient to fully eliminate vaccine-serotype circulation in the model. We show that the observed impact is likely to be sustained despite waning effects of the catch-up campaign.

## Highlights

• We predict a substantial decline in the carriage prevalence of vaccine serotypes.
• About a 56% reduction in invasive pneumococcal disease is also predicted.
• The decline is predicted to be sustainable ten years post-vaccination.
• The current vaccination schedule is unlikely to achieve elimination of vaccine serotypes.

## 1 Background

Reduction in nasopharyngeal carriage of vaccine-type pneumococci has been documented after vaccination with pneumococcal conjugate vaccines (PCVs) [1], [2], [3]. Moreover, by reducing pneumococcal acquisition, PCVs reduce pneumococcal transmission in the community offering indirect protection to the unvaccinated [4]. However, non-vaccine-type pneumococci rapidly colonise this vacated ecological niche, which can result in serotype replacement carriage [5] and replacement disease reducing the overall impact of PCVs [6]. With support from Gavi, The Vaccine Alliance, African countries have been introducing PCVs since 2009. Kenya introduced a 10-valent PCV (PCV10) targeting serotypes 1, 4, 5, 6B, 7F, 9V, 14, 18C, 19F and 23F in 2011. In Kilifi, a coastal area with enhanced surveillance for invasive pneumococcal disease (IPD) and carriage prevalence, the introduction was supplemented by a catch-up campaign in children <5 years old. At the same time annual carriage prevalence surveys have been conducted in the Kilifi Health and Demographic Surveillance System (KHDSS) population since 2009 [5].

Within a few months post-vaccination vaccine-type pneumococcal carriage and disease had dropped substantially in all age groups. However, vaccine serotypes (VTs) continue to circulate in the community [7], [5]. This raises the concern that, after the population effects of the catch-up campaign have worn off, vaccine-type pneumococcal disease will re-emerge.

We developed a dynamic compartmental model parameterized with detailed data from the KHDSS population [8] to describe the pre-vaccination pneumococcal epidemiology and predict the long-term impact of PCV10 in Kilifi. We use post-vaccination data on carriage and disease over the past five years for validation of the model predictions.

## 2 Methods

### 2.1 Data

Kilifi County Hospital (KCH) is the main referral hospital in KHDSS. At KCH, morbidity events linked with the population register have been used to define the incidence of hospital presentation with infectious diseases, including IPD [8], [9]. Datasets on pneumococcal carriage, IPD and contact patterns in KHDSS are described in detail elsewhere [5], [10], [11]. Here we briefly describe them as used in the current analysis.

#### 2.1.1 Nasopharyngeal pneumococcal carriage surveys

Two cross-sectional surveys of pneumococcal carriage were done pre-vaccination. Nasopharyngeal swabs were collected and pneumococcal serotype-specific carriage ascertained [5] to obtain the pre-vaccination age-specific prevalence and serotype distribution of carriage. The two datasets were combined since there were no significant differences between them in the carriage prevalence or serotype distribution (Appendix chapter 1).

The non-vaccine serotypes (NVTs) were classified as *weak* or *strong* based on their susceptibility to competition and carriage incidence, as estimated in a prior field study within KHDSS [12]. Strong NVTs (23B, 11A, 15A, 6A, 16F, 35B, 10A, 13, 23A 19A, 21; ordered by increasing susceptibility to competition) were less susceptible to competition. Two NVTs (34, 15B/C) were classified as strong for their higher carriage incidences compared to many of the ones chosen on the basis of susceptibility. The remaining NVTs were classified as weak (Appendix chapter 2).

#### 2.1.2 Prospective diary survey

Selected residents from KHDSS filled in a diary on the ages of all persons they physically contacted on one randomly assigned weekday [10]. For children, the diary was completed by their guardians. This information defined a social mixing matrix of contact frequencies between age groups.

### 2.2 Carriage model structure

**Fig. 1:**
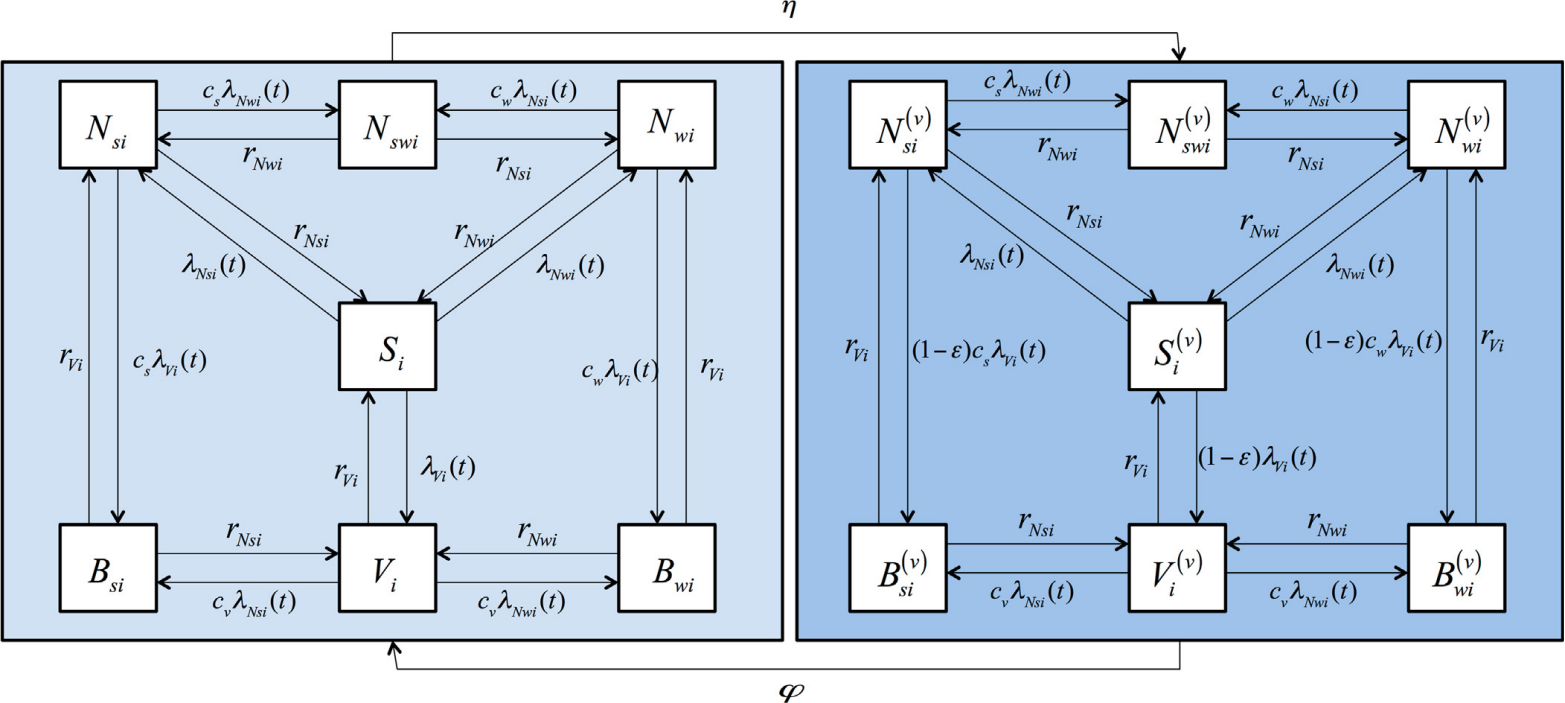
Model structure flow diagram. The epidemiological states include individuals that are susceptible (non-carrying), S; carry a vaccine serotype, V; carry a weak non-vaccine serotype, $N_{w}$; carry a strong non-vaccine serotype, $N_{s}$; carry simultaneously a weak and a strong non-vaccine serotype, $N_{\mathrm{sw}}$; carry simultaneously a vaccine serotype and a weak non-vaccine serotype, $B_{w}$; or carry simultaneously a vaccine serotype and a strong non-vaccine serotype, $B_{s}$ (see text). Once vaccinated, the individual moves to one of the corresponding states, $\left(S^{\left(v\right)}\text{,}V^{\left(v\right)}\text{,}N_{w}^{\left(v\right)}\text{,}N_{s}^{\left(v\right)}\text{,}B_{w}^{\left(v\right)}{\mathrm{andB}}_{s}^{\left(v\right)}\right)$. The acquisition rates from the single to multiple serotype carriage states are reduced by competition parameters denoted by c with two subscripts; the first denoting the serotype group (v,sandw, for VT, strong NVT and weak NVT respectively) of the resident serotypes and the second denoting the age-group. The competition parameters have two sets of values, one for age group <6 and another for age group ≥6 years (see text). The age-group specific VT, weak NVT and strong NVT clearance rates are denoted by ${\text{r}}_{\text{Vi}}\text{,}{\text{r}}_{\text{Nwi}}$ and ${\text{r}}_{\text{Nsi}}$, respectively. In addition to the transitions between the 14 epidemiological states as shown in the figure, individuals die from any states at age-specific death rates and new individuals are born into the completely susceptible state.

We developed a compartmental, age-structured dynamic model with 14 pneumococcal carriage states (Fig. 1). The model has a Susceptible-Infected-Susceptible (SIS) structure for three serotype groups: the PCV10 serotypes, strong NVT and weak NVT.

At any point in time, an unvaccinated individual can be susceptible (non-carrying) in state S; carry a VT, V; carry a weak NVT, $N_{w}$; carry a strong NVT, $N_{s}$; carry simultaneously a weak and strong NVT, $N_{\mathrm{sw}}$; carry simultaneously a VT and weak NVT, $B_{w}$; or carry simultaneously a VT and a strong NVT, $B_{s}$. Once vaccinated, the individual moves to one of the corresponding states ($S^{\left(v\right)}$, $V^{\left(v\right)}$, $N_{w}^{\left(v\right)}$, $N_{s}^{\left(v\right)}$, $N_{\mathrm{sw}}^{\left(v\right)}$, $B_{w}^{\left(v\right)}$, $B_{s}^{\left(v\right)}$). The equations of inter-state transitions are presented in Appendix chapter 3.

### 2.3 Parameterisation

#### 2.3.1 Population structure

The model population is stratified into six age groups (<1, 1–5, 6–14, 15–20, 21–49 and ⩾50 years) corresponding to those in the diary survey and reflecting the age structure in KHDSS as of 1st January 2010. Individuals in the model are born completely susceptible to carriage according to prevailing birth rates and die according to age-specific mortality rates from KHDSS (Table 1)*.*

**Table 1:** Parameters of the dynamic transmission model and the sources of information. The parameters are classified as those estimated (calibrated) in the context of the model and those derived from external sources.

Parameter/input	Estimate/value (interval^a^)	Source
*Calibrated*		
Competition parameters	$c_{s0}$ = 0.44 (0.13, 0.82) $c_{w0}$ = 0.59 (0.19, 0.96) $c_{v0}$ = 0.39 (0.15, 0.71) $c_{s}$ = 0.11 (0.004, 0.49) $c_{\mathrm{vw}}=c_{v}=c_{w}$ = 0.77 (0.30, 0.99)	Estimated

Probability of infection per 100 contacts	$q_{1}$ = 0.13 (0.07, 0.25) $q_{2}$ = 0.40 (0.30, 0.55) $q_{3}$ = 0.32 (0.24, 0.43) $q_{4}$ = 0.07 (0.04, 0.13) $q_{5}$ = 0.16 (0.11, 0.23) $q_{6}$ = 0.06 (0.04, 0.09)	Estimated
Case-to-carrier ratios^b^	Appendix chapter 7	[11]

*From external sources*		
Clearance rates	Appendix chapter 3	[12]
Birth rate	32.0 per 1000/year	[8]
Age-specific mortality	Appendix chapter 5	[8]
Contact rates		[10]
Vaccine efficacy against carriage acquisition (ε)	50% (40–60)	[3], [25], [26], [27]
Vaccine efficacy against IPD	85% (80–90)	[34]
Waning rate of protection against carriage (φ)	0.12 per year (0.09–0.20)	[35]
Routine vaccination coverage (η)	80% (70–90)	[9], [11]
Catch-up coverage	65% (60–70)	[9], [11]

a The intervals indicated for the estimated parameters are 95% credible intervals. The intervals indicated for the rest of the parameters are the ranges within which they were sampled in the model to account for their uncertainty and assess the model’s sensitivity.

b IPD incidence from Kilifi district hospital in KHDSS is divided by the carriage incidence from the model to obtain case-to-carrier ratios (Appendix chapter 7).

#### 2.3.2 Acquisition of carriage

A susceptible unvaccinated individual in age group *i* becomes colonised with VTs, strong NVTs or weak NVTs at age-group-specific time-dependent rates (forces of infection) denoted by $\lambda_{\mathrm{Vi}}(t)$, $\lambda_{\mathrm{Nsi}}(t)$ and $\lambda_{\mathrm{Nwi}}(t)$, respectively. The forces of infection were expressed as functions of the social mixing matrix and age-group specific factors ($q_{i}$) that scale the rate of social contacts into infectious contacts (Appendix chapter 3). Due to competition between serotypes in colonising the nasopharynx, the acquisition rate of a secondary serotype is lower than the acquisition rate of that serotype in a completely susceptible individual. Three competition parameters, $c_{v0}$, $c_{w0}$ and $c_{s0}$, represent the fraction by which acquisition rates of secondary serotypes are reduced in <6 year olds infected with VTs, weak NVTs and strong NVTs, respectively. Two competition parameters, $c_{\mathrm{vw}}=c_{v}=c_{w}$ and $c_{s}$, were used for individuals aged ⩾6 years infected with VTs/weak NVTs and strong NVTs, respectively.

#### 2.3.3 Clearance of carriage

The immune clearance rates of carriage (Appendix chapter 4) depend on the serotype group and age (<1, 1–5 and >5 years) and were obtained from a prior study in Kenyan children [12].

#### 2.3.4 Disease

For each serotype group and age group, case-to-carrier ratios were calculated as ratios of the observed IPD incidence at KCH [11] to the respective model-predicted pre-vaccination carriage incidence. The case-to-carrier ratios were assumed to remain unchanged post-vaccination and were multiplied with the predicted carriage incidence to predict post-vaccination IPD incidence.

#### 2.3.5 Vaccination

In Kenya, children receive PCV10 at age 6, 10 and 14 weeks. In the model, η=80% of all newborns are considered vaccinated at age 18 weeks, one month after the third dose of the 3-dose series (Table 1). A catch-up programme is simulated by vaccinating 65% of children younger than 5 years at the onset of the vaccination programme. Upon vaccination, an individual moves to the corresponding state in the vaccine-protected compartment based on his/her prevailing carriage status.

The vaccine efficacy against carriage is modelled as a 50% reduction (ε=0.50) in the acquisition rate of VTs in a vaccinated individual relative to an unvaccinated individual (Table 1). The vaccine efficacy against carriage progression to disease (VE_prog_) was calculated as a function of ε and the vaccine efficacy against IPD (VE_IPD_ = 85%) as:

$${\mathrm{VE}}_{\mathrm{prog}}=1-\frac{1-{\mathrm{VE}}_{\mathrm{IPD}}}{1-\varepsilon }=70\%\text{.}$$

We assumed that a proportion φ = 0.12 of the vaccinated population loses their protection every year. This corresponds to an average duration of protection for an individual of just over 8 years (Table 1).

#### 2.3.6 Implementation and model calibration

In the first stage, the stationary solution of the transmission model was fitted to the age-stratified pre-vaccination carriage prevalence and serotype distribution (Appendix chapter 1). Using a multinomial likelihood function and uninformative priors in a Bayesian framework, the five competition parameters ($c_{\mathrm{vw}}$, $c_{v0}$, $c_{s}$, $c_{s0}$ and $c_{w0}$) and six scaling/infectivity parameters $\left(q_{1}\text{,}q_{2}\text{,}q_{3}\text{,}q_{4}\text{,}q_{5}\text{,}q_{6}\right)$ were estimated (Appendix chapter 5). In each iteration, bootstrapping the social contact data and reconstructing the mixing matrix incorporated uncertainty in the social contact rates. A stationary population with equal birth and mortality rates was assumed.

In the second stage, the posterior samples of model parameters obtained in the first stage were applied in a prediction model. Projections were made assuming a constant population. To measure how fast the effect of the catch-up campaign wanes, we calculated the additional cases of IPD the campaign prevents in the first 10 years and estimated the time required to achieve 90% of that effect. Simulations were performed in R [13].

### 2.4 Sensitivity analysis

The sensitivity of the predicted IPD incidence averted, i.e., the difference in the overall incidence of IPD before and at 10 years post-vaccination, was assessed with respect to uncertainties in the assumed levels of: (i) vaccine efficacy against carriage acquisition; (ii) vaccine efficacy against IPD; (iii) the waning rate of vaccine-induced protection against carriage; (iv) vaccine coverage.

We performed additional simulations under a growing population using birth and death rates corresponding to the local demographics [8]. The probabilities of contact per person per day were recalculated for each time step according to the current population (Appendix chapter 6).

### 2.5 Model validation

We visually assessed proximity of the base-case predictions of the age-group specific carriage of VTs and NVTs to the corresponding observed values over a five-year period post-vaccination (2011–2015).

## 3 Results

### 3.1 Model fit to pre-vaccination epidemiology

**Fig. 2:**
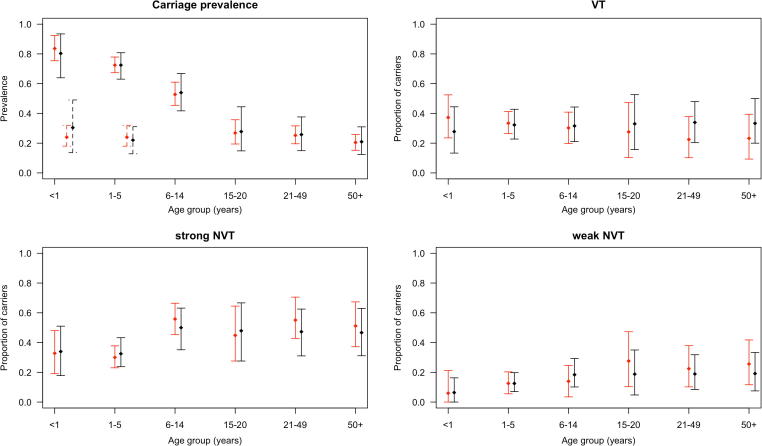
Model fit. Observed prevalence (red points) of pneumococcal carriage across age groups (top-left panel) and the proportion of carriers of VT (top-right panel), strong NVT (bottom-left panel) and weak NVT (bottom-right panel) among pneumococcal carriers prior to vaccine introduction. The black points show the corresponding estimated values of the prevalence/proportion, based on data given in Appendix chapter 1. The capped bars represent the 95% credible intervals. The dotted lines in the top-left panel (and the points they pass through) are the observed (red) and the predicted (black) proportions of double carriers among <1 and 1–5 year olds. For these two age groups, the top-right, bottom-left and bottom-right panels present the proportions of single carriers of the respective types (VT, strong NVT, weak NVT) among all carriers in the age group. (For interpretation of the references to colour in this figure legend, the reader is referred to the web version of this article.)

There was a good agreement between the observed age-group and serotype-group specific pre-vaccination carriage prevalence and their posterior estimates (Fig. 2). Within each age group, the 95% credible intervals agreed with the data. Nonetheless, the differences in the posterior mean estimates of the proportions of carriers of VTs and NVTs among pneumococcal carriers were in most instances larger than observed in individuals ≥6 years old, compared to the differences in individuals <6 years old.

### 3.2 Competition parameters

The probability of infection per contact was higher among 1–5 and 6–14 year olds as compared to other age groups (Table 1). An individual <6 years carrying a vaccine-serotype had a 61% (95% credible interval, CrI, 29–85%) protection against acquiring NVTs, relative to an uninfected individual of the same age group. In older age groups, the corresponding level of protection was 23% (95% CrI 1–70%).

### 3.3 Model projections on pneumococcal carriage

Under the base-case model, the overall prevalence of pneumococcal carriage was estimated to remain essentially at its pre-vaccination level, with only a slight reduction from 44% to 41% within 10 years post-vaccination. The prevalence of VTs in the overall population was estimated to reduce from 16% to 4%, with a simultaneous increase in the prevalence of NVTs from 28% to 36%.

**Fig. 3:**
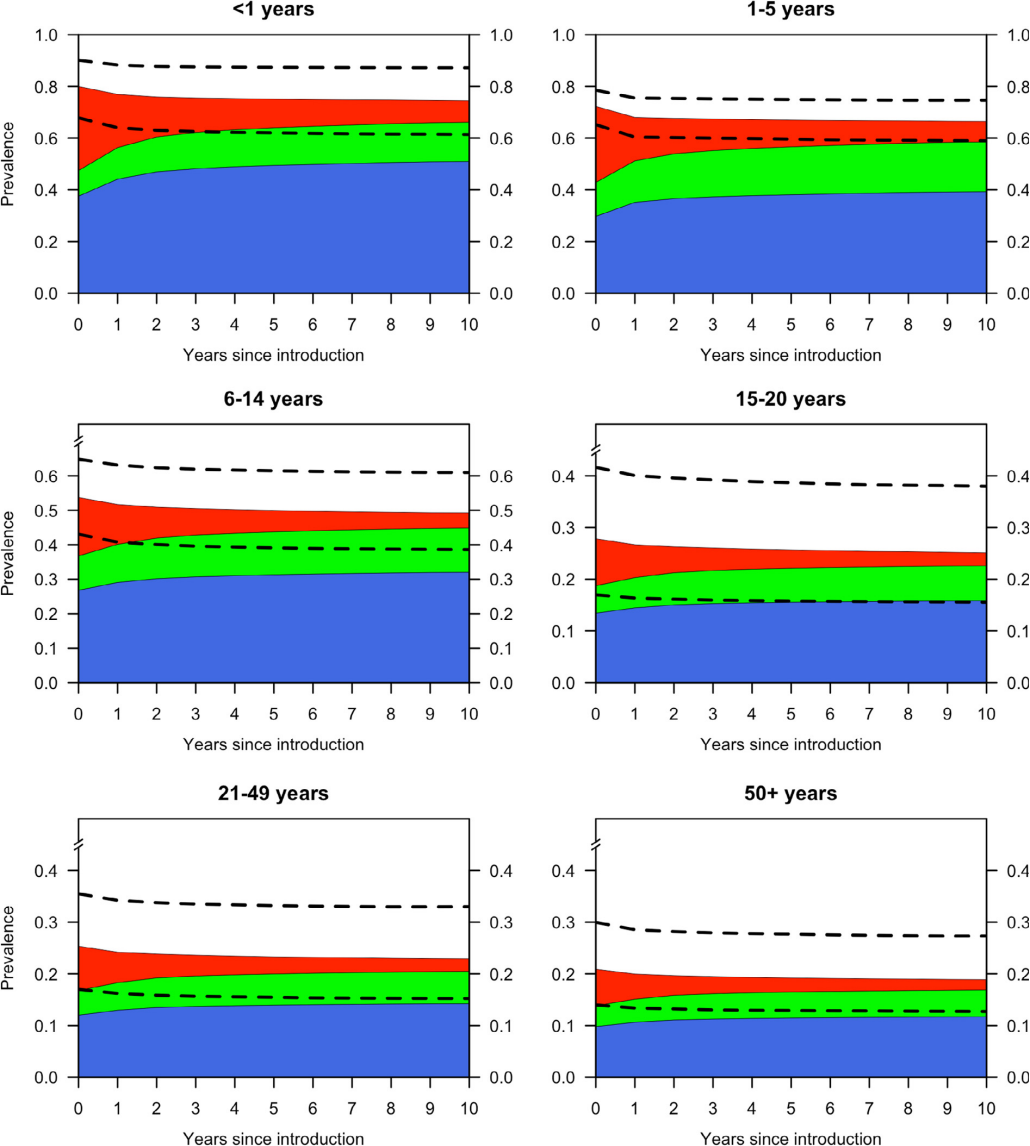
Model projections on carriage prevalence over 10 years by age group. Projected cumulative prevalence of pneumococcal carriage of VT (red), strong NVT (blue) and weak NVT (lime green) by age group over time since vaccine introduction. For each age group, the dotted lines show the 95% predictive intervals for the overall prevalence of pneumococcal carriage. (For interpretation of the references to colour in this figure legend, the reader is referred to the web version of this article.)

The prevalence of VTs was predicted to reduce in all age groups. In the older, mostly unvaccinated population, the reduction was estimated to be about two thirds of the pre-vaccination level (Table 2), suggesting a benefit of herd immunity. Changes in the prevalence of VTs and NVTs occur within the first 4–5 years post-vaccination and little change was predicted thereafter (Fig. 3).

**Table 2:** The prevalence of nasopharyngeal carriage of pneumococci pre- and 10 years post-vaccination. The table presents the posterior predictive mean values with the 95% posterior predictive intervals.

Age group (years)	Pre-vaccination				10 years post-vaccination			
	Carriage prevalence	VT^a^	Strong NVT^b^	Weak NVT	Carriage prevalence	VT	Strong NVT	Weak NVT
<1	80.8 (67.8–90.1)	32.6 (25.1–40.5)	37.5 (30.6–45.8)	9.9 (7.1–13.2)	75.7 (61.4–87.2)	8.3 (1.6–18.2)	50.9 (40.9–62.6)	15.2 (10.5–22.2)
1–5	72.5 (65.2–78.5)	29.5 (23.9–35.2)	29.7 (24.6–35.2)	13.1 (9.6–17.0)	67.2 (58.9–74.6)	8.0 (1.5–16.9)	39.2 (32.2–48.5)	19.3 (14.1–25.5)
6–14	54.0 (43.1–64.8)	17.0 (13.9–21.2)	26.8 (18.9–35.0)	9.9 (7.4–13.5)	49.7 (38.6–61.0)	4.4 (0.9–9.6)	32.0 (23.6–41.2)	12.8 (9.5–17.1)
15–20	27.9 (17.0–41.7)	9.1 (5.7–13.9)	13.4 (7.6–20.7)	5.3 (3.1–8.6)	25.3 (15.6–38.0)	2.5 (0.5–6.0)	15.8 (9.5–24.3)	6.8 (4.1–10.8)
21–49	25.5 (17.0–35.5)	8.6 (5.8–12.1)	12.0 (7.6–17.5)	4.8 (3.0–7.2)	23.2 (15.2–33.0)	2.5 (0.5–5.6)	14.2 (9.2–20.9)	6.2 (3.9–9.3)
50+	21.0 (14.0–30.0)	7.1 (4.7–10.1)	9.8 (6.2–14.6)	4.0 (2.6–6.0)	19.1 (12.7–27.3)	2.0 (0.4–4.5)	11.7 (7.6–17.2)	5.2 (3.3–7.9)

Overall	44.4 (40.2–48.9)	15.9 (13.3–18.7)	20.4 (16.8–24.2)	8.0 (6.1–10.2)	40.8 (36.0–46.0)	4.3 (0.8–9.1)	25.4 (21.2–30.0)	10.9 (8.4–13.8)

a Vaccine serotypes.

b Non-vaccine serotypes.

### 3.4 Model projections on IPD

The incidence of IPD from VTs was projected to decline in all age groups. The changes in IPD and carriage were linked and over 50% reduction in IPD occurs within the first 4–5 years after PCV introduction. The overall reduction in the incidence of IPD ten years post-vaccination is predicted to be 56% (Table 3). The overall reduction in the incidence of IPD from year 5 to 10 was 7% (95% predictive interval: −0.4% to 14%). As a result of waning direct effects of the catch-up campaign and increasing herd-effects of routine immunisation with time, we estimated that the incremental benefit of a catch-up over routine vaccination alone would be negligible from year 7 after introduction of PCV10.

**Table 3:** The incidence of invasive pneumococcal disease (IPD) pre- and 10 years post-vaccination. The table presents the posterior predictive mean values with the 95% predictive intervals.

Age group (years)	Pre-vaccination IPD incidence (/100,000/year)			10 years post-vaccination IPD incidence (/100,000/year)			
	VT	Strong NVT	Weak NVT	VT	Strong NVT	Weak NVT	IRR^a^
<1	67.0	21.0	7.1	6.7 (1.1, 15.1)	37.0 (29.5, 50.4)	11.6 (9.4, 15.6)	0.59 (0.51, 0.72)
1–5	39.3	5.1	0.7	6.4 (1.2, 14.7)	7.8 (6.5, 10.0)	1.1 (0.9, 1.4)	0.34 (0.24, 0.50)
6–14	7.3	1.0	0.0	1.4 (0.3, 3.3)	1.2 (1.1, 1.5)	0.0 (0.0, 0.0)	0.33 (0.20, 0.54)
15–20	1.0	0.0	0.0	0.3 (0.1, 0.5)	0.0 (0.0, 0.0)	0.0 (0.0, 0.0)	0.26 (0.05, 0.54)
21–49	4.2	0.9	0.0	1.2 (0.2, 2.3)	1.1 (1.0, 1.3)	0.0 (0.0, 0.0)	0.45 (0.28, 0.66)
≥50	9.2	2.8	4.1	2.5 (0.5, 5.1)	3.4 (3.1, 3.9)	5.3 (4.7, 6.3)	0.71 (0.60, 0.82)

All ages	14.8	2.7	0.9	2.5 (0.5, 5.6)	4.2 (3.5, 5.3)	1.3 (1.1, 1.6)	0.44 (0.34, 0.56)

a The incidence rate ratio (IRR) is between the overall IPD incidence before vaccination and the IPD incidence 10 years post vaccination.

### 3.5 Sensitivity analyses

**Supplementary Fig. 1:**
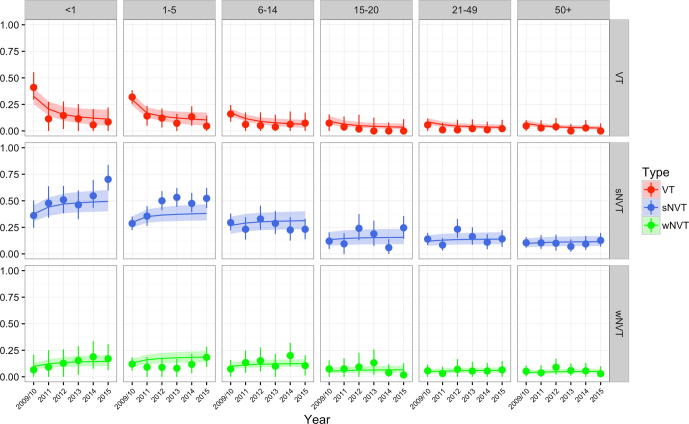
Sensitivity analysis for inputs. The impact of uncertainties in four selected model inputs (y-axis) on the predicted number of averted IPD cases per year per 100,000 in year 10 after vaccine introduction (x-axis). The vertical line at the middle of the plot marks the mean number of IPD cases (10.2) averted in year 10 per 100,000 when all the shown inputs are at their respective means. The horizontal bars correspond to predicted ranges of the number of averted IPD cases when each input variable is sampled from a normal distribution, with respective 95% ranges as given in the brackets. The number of averted cases is based on a linear model approximation of the simulation output.

Among the variables included in the sensitivity analysis, the duration of protection had the largest effect on the predicted IPD incidence averted in year 10, followed by the vaccine efficacy against carriage. The vaccine efficacy against IPD had the least influence (Fig. S1).

Assuming a growing population, the overall prevalence of carriage was projected to decline to a somewhat lower level of 35% (95% prediction interval 30–40%) ten years post-vaccination (Appendix chapter 7).

### 3.6 Model validation

**Supplementary Fig. 2:**
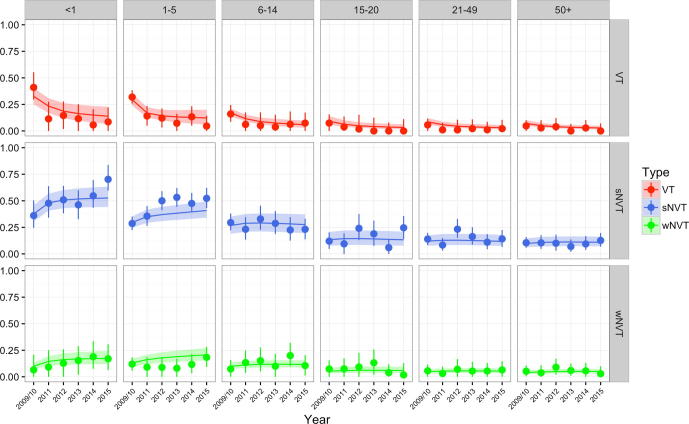
Observed and predicted carriage prevalence over time, under constant population. Observed (circular dots with 95% credible intervals shown by spikes) and predicted (lines with 95% predictive intervals shown by shaded areas) carriage prevalence of VT (red), strong NVT (blue) and weak NVT (lime green) over time since vaccine introduction. The age groups are labelled at the panel titles.

The point predictions and corresponding 95% prediction intervals (PI) of carriage prevalence cover most of the observed values, showing good predictive ability (Fig. S2). Among <1 and 1–5 year olds the model predicted much lower carriage prevalence of NVTs in year 2015 (49% vs. 70% observed and 38% vs. 52% observed, respectively).

## 4 Discussion

We used a model calibrated with local data to predict the incidence of pneumococcal carriage and IPD in Kilifi, Kenya, over a 10-year period post-vaccination to assess whether additional measures have to be considered to prevent a resurgence of vaccine-type pneumococci once the impact of the catch-up campaign wanes. We validated the model against immediate post-vaccination epidemiological data, a unique exercise in pneumococcal carriage models, and found that such resurgence is unlikely if the routine immunisation programme continues.

Most PCV introductions in African countries have occurred since year 2011. Therefore, only a few years of observation are available to assess impact. A meta-analysis of four randomized trials in African children aged 9–24 months showed that carriage of VTs decreased with vaccination but the overall carriage remained the same [14]. In the United Kingdom, the overall prevalence of pneumococcal carriage was stable four years post-vaccination [15]. In our model predictions, the overall carriage prevalence remains essentially unchanged due to serotype replacement in carriage. Replacement carriage was most prominent in <6 year olds because the pre-vaccination proportion of VTs among pneumococcal carriers was highest in young children (Appendix chapter 1). We predict that elimination of VTs in this community is unlikely. In high-income countries that have almost eliminated circulation of VTs, a reduced-dose schedule has been considered to improve the cost-effectiveness of the programme [16]. The World Health Organization (WHO) also recently convened a working group to review the policy recommendations for the optimal use of PCVs in low- and middle-income countries, which includes discussion of reduced dose schedules [17]. Theoretically, where herd protection has been established, it may be possible to sustain it using, for example, a single dose in infancy and a booster dose in the second year of life. In the Kenyan setting, however, where vaccine-type pneumococci continue to circulate several years post introduction of PCV with a catch-up campaign, it would be difficult to argue that disease prevention among infants is currently guaranteed by herd protection.

In the model presented, the incidence of IPD is predicted to decline across all age groups. The non-vaccine-type IPD incidence is expected to increase by 52%, which translates to an increase in the annual incidence of 1.9 per 100,000, suggesting little replacement disease relative to the reduction in the annual overall vaccine-type IPD incidence of 12.3 per 100,000. This is explained by the lower average case-to-carrier ratios (i.e., lower invasiveness) of the replacing non-vaccine serotypes (Appendix chapter 8).

**Supplementary Fig. 3:**
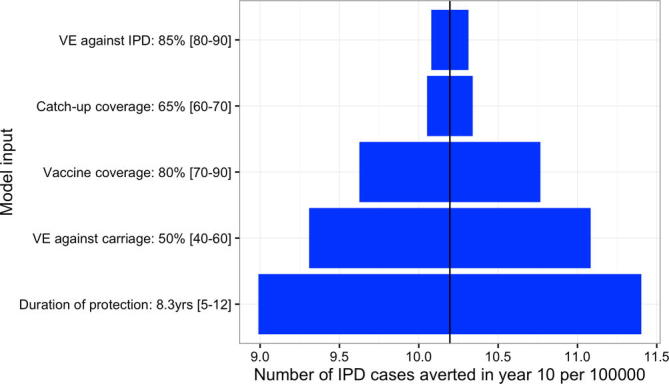
Observed and predicted carriage prevalence over time, under growing population. Observed (circular dots with 95% credible intervals shown by spikes) and predicted (lines with 95% predictive intervals shown by shaded areas) carriage prevalence of VT (red), strong NVT (blue) and weak NVT (lime green) over time since vaccine introduction. The age groups are labelled at the panel titles.

South Africa and The Gambia introduced PCV7 in 2009 and replaced it with PCV13 in 2011 [18], [19]. The reduction in vaccine-type and overall IPD reported in these countries are similar to the predictions our model produces for Kilifi, Kenya, over the first few years post-vaccination. This, however, does not validate the model because of differences across the settings. The vaccination coverage in Kenya is likely to differ from coverage in The Gambia and South Africa, and Kenya introduced PCV10. We thus validated our model predictions against observed carriage prevalence and IPD incidence in Kilifi. The model predictions were generally consistent with the observed data (Fig. S2). The model, however, underestimated prevalence of carriage of NVTs in <6 year olds in 2014–15. Relaxing the assumption of a constant population size only made minimal difference to the goodness of fit (Fig. S3).

Pneumococcal serotypes are heterogeneous in transmissibility and mutual competition [12], [20]. By splitting the NVTs into two groups and allowing unequal mutual competition between these groups, our model accounts for some of this heterogeneity. We did not split VTs because we aimed to reproduce serotype replacement with as small a number of parameters as possible, by limiting the number of compartments. Splitting NVTs was preferred because the group has a larger number of serotypes and hence more heterogeneity. The model projected differing magnitudes of change in the prevalence of the strong and weak NVTs. Given the different case-to-carrier ratios of the two groups of NVTs (Appendix chapter 8), the projected non-vaccine-type IPD incidence is different from what would have been predicted using a single group of NVTs. Nonetheless, grouping serotypes can create some ‘super types’ that might have different characteristics, e.g. higher acquisition rate of the VTs group compared to the individual serotypes in the group. This might lead to conservative vaccine effectiveness estimates. Grouping of serotypes may also result in the estimated acquisition rate of NVTs being lower than that of individual serotypes in the group. This would lead to an underestimation of the indirect impact of vaccination on NVTs - lower than the observed predicted prevalence of NVTs.

To limit the number of estimated parameters, age dependency in competition was considered using two age classes (<6 and ⩾6 years). Some discrepancies between the fitted and observed age-specific serotype distributions were present. The proportion of carriers of VTs was overestimated among carriers aged ≥15 years (Fig. 2); the susceptibility to competition of VTs against NVTs is likely biased downwards in adults, thus underestimating the reduction in prevalence of VTs. With our current specification, the estimates of competition parameters in age group ≥6 years largely depends on data from the age groups 6–14 years. A model including more groups of VTs and NVTs or individual serotypes [21], [22], [23] would allow for even more heterogeneity. However, the estimation of competition parameters from available carriage data would become increasingly difficult in a highly compartmentalized model.

We estimated case-to-carrier ratios using hospital-based data on IPD incidence in KHDSS [8]. The access to care for IPD is unknown in KHDSS, but meningitis incidence is underestimated by over 30% by hospital-based surveillance [24]. Since IPD and meningitis are severe syndromes, the underestimation of IPD incidence could be similar implying case-to-carrier ratios are likely underestimated. Nonetheless, since the ratios estimated pre-vaccination are applied post-vaccination, the predicted reduction in IPD is not affected.

We excluded partial protection from first and second doses. Our estimates of the vaccine impact may thus be conservative if the vaccines’ efficacy is substantial after fewer than three doses. We treated vaccine efficacy against carriage and its waning as equal for routine and catch-up vaccination. A Kenyan trial estimated vaccine efficacy against carriage of 40% among children aged 1–4 years [3], lower than the 50% for infant vaccination [25], [26], [27]. The duration of protection of catch-up vaccination is not documented yet. One dose of PCV administered outside of infancy may have a more enduring effect than 3 routine infant doses. If so, our similar treatment of the duration of immunity means there is no inflection on the carriage prevalence of VTs as the cohort of highly immune <5 year olds who received a catch-up dose is replaced by a new birth cohort of less immune children over time.

We assumed children are born completely susceptible to acquisition of pneumococcus ignoring the influence of maternal antibodies. Newborns in a Kenyan study had a very high rate of first acquisition [20]. Early acquisition has also been reported in other African settings [28], [29], [30]. In Netherlands and Papua New Guinea a protective effect of maternal IgG antibodies against colonisation in infancy was not observed [31], [32]. Based on high early acquisition rates and insufficient evidence of protection from maternal antibodies in some studies, this assumption is plausible.

A significant reduction in IPD caused by vaccine-related serotypes 6A and 19A IPD has been observed in some PCV10-using settings [33]. However, surveillance in Kilifi recorded no change in carriage of serotype 6A and increased carriage of serotype 19A after vaccine introduction [7]. We have not observed a change in IPD caused by these serotypes. We therefore did not account for 6A and 19A as vaccine serotypes.

In conclusion, we predict a substantial and sustainable decline in the carriage prevalence of VTs among vaccinated and unvaccinated individuals and consequently a reduction of about 56% in overall IPD incidence ten years post-vaccination. While we show that the current schedule is sufficient to limit vaccine-type pneumococcal carriage to current levels, it is unlikely to achieve elimination of VTs. Strategies that heavily rely on protection from the herd, including a reduced dose schedule, will need additional efforts to stop circulation of VTs before their implementation.

## Author contributions

Conceived the study: JO, KA, MN, JAGS, SF. Model coding and simulations: JO*.* Conducted the pneumococcal carriage surveys and/or facilitated IPD surveillance in Kilifi: LLH, DA, IA, JAGS, TK. Conducted the social contact survey: MCK. All authors: read and appraised the scientific content of the manuscript.

## Ethics statement

The study was part of the Pneumococcal Conjugate Vaccine Impact Study (PCVIS) approved by the Kenya Medical Research Institute (KEMRI) Ethical review committee (SCC 1433). It has an additional approval by OXTREC (OXTREX 30-10), the Oxford Tropical Research Ethics Committee, with delegated authority from the London School of Hygiene & Tropical Medicine (LSHTM) Research Ethics Committee.

## Conflict of interest

None.
