# Supplementary material for: Sustained reduction in vaccine-type invasive pneumococcal disease despite waning effects of a catch-up campaign in Kilifi, Kenya: A mathematical model based on pre-vaccination data
Source: Vaccine. 2017 Aug 16;35(35Part B):4561–8. doi: 10.1016/j.vaccine.2017.07.019 (PMC5571446; doi:10.1016/j.vaccine.2017.07.019)
Supplement: Supplementary Appendix [file mmc1.docx]

**Appendix**

**Chapter 1. Prevalence of pneumococcal carriage in Kilifi, Kenya.**

Serotypes were grouped as vaccine-types (VT, those included in PCV10), strong non-vaccines serotypes (23B, 11A, 15A, 6A, 16F, 35B, 10A, 13, 23A, 19A, 21, 34, 15B/C; see appendix chapter 2), and weak non-vaccine-types (the rest). The data came from two surveys of pneumococcal carriage in Kilifi, Kenya (2009-2010) [5]. The reported prevalences in table A.1 below are age-standardised, with the KHDSS reference population as at 1 Jan 2010 (midpoint of the two survey years), since the surveys were based on 10 age strata collapsed to six in the current analysis. Based on the total number of individuals in each age group, the numbers of carriers of VTs, strong NVTs and weak NVTs as presented in this table were calculated to match the estimated standardised carriage prevalence of the respective serotype groups.

**Table A.1:** Prevalence of pneumococcal carriage and the serotype distribution by age group.

| Age group  (years) | Number of carriers N (%) | VT  N (%) | Strong NVT  N (%) | Weak NVT  N (%) | Total  N (%) |
| --- | --- | --- | --- | --- | --- |
|  |  |  |  |  |  |
| <1 | 51 (83.6) | 25 (41.0) | 22 (36.1) | 4 (6.6) | 61 (100.0) |
| 1-5 | 200 (72.5) | 88 (31.9) | 79 (28.6) | 33 (12.0) | 276 (100.0) |
| 6-14 | 86 (52.8) | 26 (16.0) | 48 (29.4) | 12 (7.4) | 163 (100.0) |
| 15-20 | 29 (26.9) | 8 (7.4) | 13 (12.0) | 8 (7.4) | 108 (100.0) |
| 21-49 | 49 (25.3) | 11 (5.7) | 27 (13.9) | 11 (5.7) | 194 (100.0) |
| ≥50 | 43 (20.5) | 10 (4.8) | 22 (10.5) | 11 (5.2) | 210 (100.0) |

*.*

**Chapter 2. Division of serotypes into VTs, strong NVTs and weak NVTs**

Vaccine serotypes (VTs) are serotype in the PCV-10 vaccine (1, 4, 5, 6B, 7F, 9V, 14, 18C, 19F and 23F). The non-vaccine serotypes (NVTs) were classified as *weak* or *strong* based on their susceptibility to competition (a measure of competitive strength of a serotype defined as the rate at which individuals carrying that serotype switch to carry another serotype, relative to the rate at which that other serotype colonizes an uncolonized person) and carriage incidence, as estimated in a prior field study within KHDSS^1^. In our model strong NVTs were categorised as those less susceptible to competition by having a lower susceptibility; NVTs with susceptibility estimate of 1 and below were considered strong. These serotypes were: 23B, 11A, 15A, 6A, 16F, 35B, 10A, 13, 23A 19A and 21, ordered by increasing susceptibility to competition. Two NVTs (34, 15B/C) were also classified as strong for their higher carriage incidences than many of the ones chosen on the basis of susceptibility. The remaining NVTs were classified as weak (Table A.2).

**Table A.2**: Categorisation of serotypes

| Serotype | Serotype  group | Susceptibility | Incidence  (per 1000 days) | Duration of carriage  (days) |
| --- | --- | --- | --- | --- |
| 19F | VTs | 0.481 | 3.07 | 88.5 |
| 23B | strong NVTs | 0.523 | 0.93 | 60.3 |
| 6B | VTs | 0.524 | 1.77 | 115.6 |
| 11A | strong NVTs | 0.544 | 1.08 | 72.3 |
| 15A | strong NVTs | 0.560 | 0.65 | 55.0 |
| 6A | strong NVTs | 0.580 | 2.51 | 123.8 |
| 23F | VTs | 0.586 | 1.46 | 67.8 |
| 16F | strong NVTs | 0.703 | 0.44 | 52.5 |
| 9V | VTs | 0.752 | 0.88 | 44.6 |
| 35B | strong NVTs | 0.813 | 1.15 | 88.1 |
| 10A | strong NVTs | 0.874 | 0.97 | 65.2 |
| 23A | strong NVTs | 0.910 | 0.34 | 54.2 |
| 14 | VTs | 0.911 | 1.36 | 69.5 |
| 19A | strong NVTs | 0.956 | 0.87 | 58.9 |
| 13 | strong NVTs | 0.969 | 0.79 | 70.4 |
| 18C | VTs | 0.970 | 0.49 | 49.7 |
| 21 | strong NVTs | 1.059 | 0.31 | 93.0 |
| 34 | strong NVTs | 1.166 | 0.71 | 76.3 |
| 19B | weak NVTs | 1.176 | 0.54 | 63.5 |
| 1 | VTs | 1.183 | 0.25 | 31.0 |
| 35A | weak NVTs | 1.191 | 0.29 | 61.9 |
| 15B | strong NVTs | 1.353 | 1.25 | 51.8 |
| other | weak NVTs | 1.366 | 1.86 | 41.3 |
| 7C | weak NVTs | 1.400 | 0.62 | 40.1 |
| 15C | strong NVTs | 1.503 | 1.08 | 56.3 |
| 3 | weak NVTs | 1.519 | 0.74 | 40.8 |
| 20 | weak NVTs | 1.533 | 0.59 | 28.3 |
| 33B | weak NVTs | 2.074 | 0.38 | 29.6 |

^1^ Lipsitch M, Abdullahi O, Dʼamour A, Xie W, Weinberger DM, Tchetgen ET, et al. Estimating Rates of Carriage Acquisition and Clearance and Competitive Ability for Pneumococcal Serotypes in Kenya With a Markov Transition Mode. Epidemiology 2012;23:1–10.

**Chapter 3. Dynamic model structure in equations**

All parameters and states in the equations below have been defined in the main article (see also Figure 1). The quantities $b,\mu_{i}$ and $P\left( t \right)$ denote the birth rate, age-specific death rate and population size at time $t$, respectively. The term $k_{i}(t)$ denotes the rate of movement to the next age group from the $i^{th}$ age group (see *Appendix chapter 6*). The term $b*P\left( t \right)$ in the first equation applies only to the first age group (< 1 year olds). In the first age group, all terms with ${+k}_{i-1}\left( t \right)$ denoting movement into the age group, by aging, from a lower age category should be excluded from all equations. In the last age group, ≥50 years, all terms with ${-k}_{i}\left( t \right)$ denoting movement from the age group by aging should be excluded from all equations.

$$\frac{dS_{i}\left( t \right)}{dt}=r_{Vi}*V_{i}\left( t \right)+r_{Nsi}*N_{si}\left( t \right)+r_{Nwi}*N_{wi}\left( t \right)-S_{i}\left( t \right)*\left( \lambda_{Vi}\left( t \right)+\lambda_{Nsi}\left( t \right)+\lambda_{Nwi}\left( t \right) \right)-\eta*S_{i}\left( t \right)+\varphi*S_{i}^{\left( v \right)}\left( t \right)+b*P\left( t \right)-\mu_{i}*S_{i}\left( t \right)-k_{i}\left( t \right)*S_{i}\left( t \right)+k_{i-1}\left( t \right)*S_{i-1}\left( t \right)$$

$$\frac{dV_{i}\left( t \right)}{dt}=r_{Nsi}*B_{si}\left( t \right)+r_{Nwi}*B_{wi}\left( t \right)-r_{Vi}*V_{i}\left( t \right)+\lambda_{Vi}\left( t \right)*S_{i}\left( t \right)-V_{i}\left( t \right)*\left( c_{v}*\lambda_{Nsi}\left( t \right)+c_{v}*\lambda_{Nwi}\left( t \right) \right)-\eta*V_{i}\left( t \right)+\varphi*V_{i}^{\left( v \right)}\left( t \right)-\mu_{i}*V_{i}\left( t \right)-k_{i}\left( t \right)*V_{i}\left( t \right)+k_{i-1}\left( t \right)*V_{i-1}\left( t \right)$$

$$\frac{dN_{si}\left( t \right)}{dt}=r_{Nwi}*N_{swi}\left( t \right)+r_{Vi}*B_{si}\left( t \right)-r_{Nsi}*N_{si}\left( t \right)+\lambda_{Nsi}\left( t \right)*S_{i}\left( t \right)-N_{si}\left( t \right)*\left( c_{s}*\lambda_{Vi}\left( t \right)+c_{s}*\lambda_{Nwi}\left( t \right) \right)-\eta*N_{si}\left( t \right)+\varphi*N_{si}^{\left( v \right)}\left( t \right)-\mu_{i}*N_{si}\left( t \right)-k_{i}\left( t \right)*N_{si}\left( t \right)+k_{i-1}\left( t \right)*N_{si-1}\left( t \right)$$

$$\frac{dN_{wi}\left( t \right)}{dt}=r_{Nsi}*N_{swi}\left( t \right)+r_{Vi}*B_{wi}\left( t \right)-r_{Nwi}*N_{wi}\left( t \right)+\lambda_{Nwi}\left( t \right)*S_{i}\left( t \right)-N_{wi}\left( t \right)*\left( c_{w}*\lambda_{Vi}\left( t \right)+c_{w}*\lambda_{Nsi}\left( t \right) \right)-\eta*N_{wi}\left( t \right)+\varphi*N_{wi}^{\left( v \right)}\left( t \right)-\mu_{i}*N_{wi}\left( t \right)-k_{i}\left( t \right)*N_{wi}\left( t \right)+k_{i-1}\left( t \right)*N_{wi-1}\left( t \right)$$

$$\frac{dN_{swi}\left( t \right)}{dt}=c_{w}*\lambda_{Nsi}\left( t \right)*N_{wi}\left( t \right)+c_{s}*\lambda_{Nwi}\left( t \right)*N_{si}\left( t \right)-\left( r_{Nsi}+r_{Nwi} \right)*N_{swi}\left( t \right)-\eta*N_{swi}\left( t \right)+\varphi*N_{swi}^{\left( v \right)}\left( t \right)-\mu_{i}*N_{swi}\left( t \right)-k_{i}\left( t \right)*N_{swi}\left( t \right)+k_{i-1}\left( t \right)*N_{swi-1}\left( t \right)$$

$$\frac{dB_{si}\left( t \right)}{dt}=c_{v}*\lambda_{Nsi}\left( t \right)*V_{i}\left( t \right)+c_{s}*\lambda_{Vi}\left( t \right)*N_{si}\left( t \right)-\left( r_{Nsi}+r_{Vi} \right)*B_{si}\left( t \right)-\eta*B_{si}\left( t \right)+\varphi*B_{si}^{\left( v \right)}\left( t \right)-\mu_{i}*B_{si}\left( t \right)-k_{i}\left( t \right)*B_{si}\left( t \right)+k_{i-1}\left( t \right)*B_{si-1}\left( t \right)$$

$$\frac{dB_{wi}\left( t \right)}{dt}=c_{v}*\lambda_{Nwi}\left( t \right)*V_{i}\left( t \right)+c_{w}*\lambda_{Vi}\left( t \right)*N_{wi}\left( t \right)-\left( r_{Nwi}+r_{Vi} \right)*B_{wi}\left( t \right)-\eta*B_{wi}\left( t \right)+\varphi*B_{wi}^{\left( v \right)}\left( t \right)-\mu_{i}*B_{wi}\left( t \right)-k_{i}\left( t \right)*B_{wi}\left( t \right)+k_{i-1}\left( t \right)*B_{wi-1}\left( t \right)$$

$$\frac{dS_{i}^{\left( v \right)}\left( t \right)}{dt}=r_{Vi}*V_{i}^{\left( v \right)}\left( t \right)+r_{Nsi}*N_{si}^{\left( v \right)}\left( t \right)+r_{Nwi}*N_{wi}^{\left( v \right)}\left( t \right)-S_{i}^{\left( v \right)}\left( t \right)*\left( {\left( 1-\varepsilon\right)*\lambda}_{Vi}\left( t \right)+\lambda_{Nsi}\left( t \right)+\lambda_{Nwi}\left( t \right) \right)+\eta*S_{i}\left( t \right)-\varphi*S_{i}^{\left( v \right)}\left( t \right)-\mu_{i}*S_{i}^{\left( v \right)}\left( t \right)-k_{i}\left( t \right)*S_{i}^{\left( v \right)}\left( t \right)+k_{i-1}\left( t \right)*S_{i-1}^{\left( v \right)}\left( t \right)$$

$$\frac{dV_{i}^{\left( v \right)}\left( t \right)}{dt}=r_{Nsi}*B_{si}^{\left( v \right)}\left( t \right)+r_{Nwi}*B_{si}^{\left( v \right)}\left( t \right)-r_{Vi}*V_{i}^{\left( v \right)}\left( t \right)+{\left( 1-\varepsilon\right)*\lambda}_{Vi}\left( t \right)*S_{i}^{\left( v \right)}\left( t \right)-V_{i}^{\left( v \right)}\left( t \right)*\left( c_{v}*\lambda_{Nsi}\left( t \right)+c_{v}*\lambda_{Nwi}\left( t \right) \right)+\eta*V_{i}^{\left( v \right)}\left( t \right)-\varphi*V_{i}^{\left( v \right)}\left( t \right)-\mu_{i}*V_{i}^{\left( v \right)}\left( t \right)-k_{i}\left( t \right)*V_{i}^{\left( v \right)}\left( t \right)+k_{i-1}\left( t \right)*V_{i-1}^{\left( v \right)}\left( t \right)$$

$$\frac{dN_{si}^{\left( v \right)}\left( t \right)}{dt}=r_{Nwi}*N_{swi}^{\left( v \right)}\left( t \right)+r_{Vi}*B_{si}^{\left( v \right)}\left( t \right)-r_{Nsi}*N_{si}^{\left( v \right)}\left( t \right)+\lambda_{Nsi}\left( t \right)*S_{i}^{\left( v \right)}\left( t \right)-N_{si}^{\left( v \right)}\left( t \right)*\left( \left( 1-\varepsilon\right)*c_{s}*\lambda_{Vi}\left( t \right)+c_{s}*\lambda_{Nwi}\left( t \right) \right)+\eta*N_{si}\left( t \right)-\varphi*N_{si}^{\left( v \right)}\left( t \right)-\mu_{i}*N_{si}^{\left( v \right)}\left( t \right)-k_{i}\left( t \right)*N_{si}^{\left( v \right)}\left( t \right)+k_{i-1}\left( t \right)*N_{si-1}^{\left( v \right)}\left( t \right)$$

$$\frac{dN_{wi}^{\left( v \right)}\left( t \right)}{dt}=r_{Nsi}*N_{swi}^{\left( v \right)}\left( t \right)+r_{Vi}*B_{wi}^{\left( v \right)}\left( t \right)-r_{Nwi}*N_{wi}^{\left( v \right)}\left( t \right)+\lambda_{Nwi}\left( t \right)*S_{i}^{\left( v \right)}\left( t \right)-N_{wi}^{\left( v \right)}\left( t \right)*\left( {\left( 1-\varepsilon\right)*c}_{w}*\lambda_{Vi}\left( t \right)+c_{w}*\lambda_{Nsi}\left( t \right) \right)+\eta*N_{wi}\left( t \right)-\varphi*N_{wi}^{\left( v \right)}\left( t \right)-\mu_{i}*N_{wi}^{\left( v \right)}\left( t \right)-k_{i}\left( t \right)*N_{wi}^{\left( v \right)}\left( t \right)+k_{i-1}\left( t \right)*N_{wi-1}^{\left( v \right)}\left( t \right)$$

$$\frac{dN_{swi}^{\left( v \right)}\left( t \right)}{dt}=c_{w}*\lambda_{Nsi}\left( t \right)*N_{wi}^{\left( v \right)}\left( t \right)+c_{s}*\lambda_{Nwi}\left( t \right)*N_{si}^{\left( v \right)}\left( t \right)-\left( r_{Nsi}+r_{Nwi} \right)*N_{swi}^{\left( v \right)}\left( t \right)+\eta*N_{swi}\left( t \right)-\varphi*N_{swi}^{\left( v \right)}\left( t \right)-\mu_{i}*N_{swi}^{\left( v \right)}\left( t \right)-k_{i}\left( t \right)*N_{swi}^{\left( v \right)}\left( t \right)+k_{i-1}\left( t \right)*N_{swi-1}^{\left( v \right)}\left( t \right)$$

$$\frac{dB_{si}^{\left( v \right)}\left( t \right)}{dt}=c_{v}*\lambda_{Nsi}\left( t \right)*V_{i}^{\left( v \right)}\left( t \right)+{\left( 1-\varepsilon\right)*c}_{s}*\lambda_{Vi}\left( t \right)*N_{si}^{\left( v \right)}\left( t \right)-\left( r_{Nsi}+r_{Vi} \right)*B_{si}^{\left( v \right)}\left( t \right)+\eta*B_{si}\left( t \right)-\varphi*B_{si}^{\left( v \right)}\left( t \right)-\mu_{i}*B_{si}^{\left( v \right)}\left( t \right)-k_{i}\left( t \right)*B_{si}^{\left( v \right)}\left( t \right)+k_{i-1}\left( t \right)*B_{si-1}^{\left( v \right)}\left( t \right)$$

$$\frac{dB_{wi}^{\left( v \right)}\left( t \right)}{dt}=c_{v}*\lambda_{Nwi}\left( t \right)*V_{i}^{\left( v \right)}\left( t \right)+{\left( 1-\varepsilon\right)*c}_{w}*\lambda_{Vi}\left( t \right)*N_{wi}^{\left( v \right)}\left( t \right)-\left( r_{Nwi}+r_{Vi} \right)*B_{wi}^{\left( v \right)}\left( t \right)+\eta*B_{wi}\left( t \right)-\varphi*B_{wi}^{\left( v \right)}\left( t \right)-\mu_{i}*B_{wi}^{\left( v \right)}\left( t \right)-k_{i}\left( t \right)*B_{wi}^{\left( v \right)}\left( t \right)+k_{i-1}\left( t \right)*B_{wi-1}^{\left( v \right)}\left( t \right)$$

The forces of infection by VTs, weak NVTs, and strong NVTs are defined by equations 1, 2 and 3 below:

$$\lambda_{Vi}\left( t \right)=\sum_{j} \beta_{ij}*\left( V_{j}\left( t \right)+B_{sj}\left( t \right)+B_{wj}\left( t \right)+V_{j}^{\left( v \right)}\left( t \right)+B_{sj}^{\left( v \right)}\left( t \right)+B_{wj}^{\left( v \right)}\left( t \right) \right) (1)$$

$$\lambda_{Nwi}\left( t \right)=\sum_{j} \beta_{ij}*\left( N_{wj}\left( t \right)+N_{swj}\left( t \right)+B_{wj}\left( t \right)+N_{wj}^{\left( v \right)}\left( t \right)+N_{swj}^{\left( v \right)}\left( t \right)+B_{wj}^{\left( v \right)}\left( t \right) \right) (2)$$

$$\lambda_{Nsi}\left( t \right)=\sum_{j} \beta_{ij}*\left( N_{sj}\left( t \right)+N_{swj}\left( t \right)+B_{sj}\left( t \right)+N_{sj}^{\left( v \right)}\left( t \right)+N_{swj}^{\left( v \right)}\left( t \right)+B_{sj}^{\left( v \right)}\left( t \right) \right) (3)$$

where the $\beta_{ij}$ is the per capita transmission rate between an individual (carrier) in age class *j* and a (susceptible) individual in age class *i*. These rates are expressed as a function of the social mixing matrix $U_{ij}$ as $\beta_{ij}=\frac{q_{i}*U_{ij}}{y_{i}}$, where the age group specific proportionality factor $q_{i}$ scales the rate of social contacts into infectious contacts and represents the susceptibility to acquisition of carriage, given a contact [36]. The elements of the matrix $U_{ij}$ are the mean numbers of social contacts an individual in age class $j$ makes with individuals in age class $i$ per unit time and $y_{i}$ is the population size of the $i^{th}$ age group. The unknown scaling factors $q_{i}$ were allowed differ across age groups, based on the initial observation that the pre-vaccination prevalence of carriage in the three first age groups was significantly different but the prevalence in each of the age groups above the age of 15 years were similar despite the varying average rates of social contacts.

**Chapter 4. Clearance rates**

To obtain the average age-group specific clearance rate for each serotype group, a weighted mean of the clearance rates of individual serotypes in the group was computed. The incidences of the individual serotypes were used as weights. For infants, the clearance rates were computed for the strong NVTs and weak NVTs. For 1-5 and ≥6 year olds, a single clearance rate was computed for the weak and strong NVTs by taking the weighted average of the clearance rates of all NVTs.

**Table A.3:** Clearance rates (per month) by age group and serotype group.

|  | Serotype group | | |
| --- | --- | --- | --- |
| Age group (years) | **VT** | **Strong NVT** | **Weak NVT** |
| <1 | 0.271 | 0.285 | 0.601 |
| 1-5 | 0.546 | 0.662 | 0.662 |
| ≥6 | 0.934 | 0.928 | 0.928 |

**Chapter 5. Estimation of the model parameters**

The model was calibrated to the age-specific prevalence and serotype distributions in the pre-vaccination era (Appendix chapter 1), assuming a steady-state distribution of carriage in the population. Since the calibration data recorded only one serotype for each carrying individual, there were only four carriage states $(S_{i}, V_{i},N_{si}\mathrm{and}N_{wi})$ on which to define the likelihood. The model output has three additional states $\left( N_{swi}, B_{si}, B_{wi} \right)$. Therefore, for the age groups 6-14, 15-20, 21-49 and ≥50 years, the model output for the seven carriage states was collapsed into four states as follows:

$$V_{i}^{'}=V_{i}+0.5*\left( B_{si}+B_{wi} \right)$$

$$N_{si}^{'}=N_{si}+0.5*\left( B_{si}+N_{swi} \right)$$

$$N_{wi}^{'}=N_{wi}+0.5*\left( B_{wi}+N_{swi} \right)$$

This choice carries the assumption that in a doubly-colonised individual either of the two colonising serotypes was detected with 50% probability.

For the two youngest age groups, <1 and 1-5 years, data on the proportion of doubly-colonised individuals among pneumococcal carriers were available from a study in Kenya. Consequently, in the numerical estimation algorithm (see below), the proportion $\rho$ “of doubly-colonised individuals in <6 year olds was randomly generated from a normal distribution with mean 24% and standard deviation of 3.5%. For any realisation of the proportion, the expected number of doubly-colonised individuals in the first two age groups is then $D_{obs}= \rho*(V+ N_{s}+ N_{w})$. The observed data for the two youngest age groups was adjusted to include five classes of carriage by

$$X_{i}=\left\{ S_{i},V_{i}\left( 1-\rho\right),N_{si}\left( 1-\rho\right),N_{wi}\left( 1-\rho\right),D_{obs i} \right\}$$

Accordingly, the model output for the carriage states was collapsed into 5 states by adding together the doubly-colonised states $\left( D_{i}=B_{si}+B_{wi}+N_{swi} \right)$ so that collapsed model output was $\left\{ S_{i}, V_{i},N_{si},N_{wi},D_{i} \right\}$ for <6 year olds.

Denote the vectors containing the number of individuals in each $j^{th}$ carriage status in the $i^{th}$ age group in the empirical calibration data by $X_{ij}$. Denote the vectors containing the model output of the proportions of the carriage status in the $i^{th}$age group by $P_{i}\left( \theta\right)$. Vector $\theta$ contains all model parameters that are estimated from the data*.*

Denote the set of model parameters by $\theta=\left\{ {q_{1},q_{2},q_{3},q_{4},q_{5},q_{6},c}_{v0},c_{s0},c_{w0},c_{vw},c_{s} \right\}$, where $q_{i}(i=1,2,3,4,5,6)$ are the proportionality factors that scale the rate of social contacts into infectious contacts and $c_{v0},c_{s0},c_{w0},c_{vw},c_{s}$ are the competition parameters. The likelihood function of the model parameters is based on a multinomial distribution. In particular, the counts $X_{i}.$ in the $i^{th}$ age group follow a multinomial distribution, so that their log-likelihood based on the observations is

$\sum_{i=1}^{6} \sum_{j=1}^{k_{i}} X_{ij}log\left( P_{ij}(\theta) \right)$,

where $k_{i}$ = 5 for the two youngest age groups and 4 otherwise. For any given set,$\theta$, the model equations (Appendix chapter 3) were solved to find the stationary numbers of individuals in each of the pre-vaccination compartments, from these numbers the age group specific carriage distribution in the four (or five for the first two age groups) compartments was derived as the model output. The routine BBsolve in R[37] was used to solve the system of equations for a stationary solution.

The Metropolis-Hastings algorithm was used to draw samples from the posterior distributions of the parameters. A non-informative (uniform distribution in the range 0-1) prior was used for each parameter and the posterior distributions of the parameters were summarised to obtain point estimates (posterior mean) and probability (credibility) intervals for parameters included in $\theta$. 100,000 MCMC iterations were used. After a burn-in of 40,000 the remaining samples, which were stationary, were thinned to 2000; the posterior means and 95% credible intervals of the parameters were calculated from these samples.

**Chapter 6. Population model**

***Population under constant mortality across age groups***

Denote the age-specific mortality, i.e., the per capita rate of death by $\upsilon\left( a \right)$. The steady-state age distribution is $f\left( a \right)=\frac{exp\left( -\int_{0}^{a} \upsilon\left( u \right)du \right)}{A}$, where the normalising constant $A=\int_{0}^{\infty} exp\left( -\int_{0}^{a} \upsilon\left( u \right)du \right)da$.

In case of constant mortality across age groups $\upsilon\left( a \right)=\upsilon$ and the steady state age distribution is exponential, i.e., $f\left( a \right)=\upsilon*exp(-\upsilon*a)$. Denote the age group boundaries $t=\left( t_{1},\ldots{,t}_{7} \right)=(0,1,6,15,21,50,\infty)$ (years) and the observed numbers of individuals in each of the age groups in the KHDSS in the year before vaccine introduction as $N_{i}=(N_{1},\ldots,N_{6})$. To estimate a constant rate $\upsilon$, the steady-state age distribution was fitted to the observed data based on a multinomial likelihood for $\upsilon$:

$$\prod_{i=1}^{6} \left[ exp(-\upsilon t_{i})-exp(-\upsilon t_{i+1}) \right]^{N_{i}}.$$

The maximum likelihood estimate for $\upsilon$ was found to be 0.046 per year (or 1.25E-04 per day). Based on this estimate, the fitted proportion of individuals in each of the age groups is shown in Table A.4.

Table A.4: Observed and model age distribution in the KDHSS before vaccine introduction. A constant birth and death rate was assumed.

| Age Group  (years) | Number of  individuals | Observed  Proportion | Model  Proportion |
| --- | --- | --- | --- |
| <1 | 9,424 | 0.037 | 0.045 |
| 1-5 | 45,727 | 0.180 | 0.195 |
| 6-14 | 68,648 | 0.271 | 0.256 |
| 15-20 | 32,815 | 0.129 | 0.121 |
| 21-49 | 72,705 | 0.287 | 0.281 |
| ≥50 | 24,288 | 0.096 | 0.102 |

Under the assumptions of a constant death rate, in age group $i$ with upper bound $a_{i}=\left( a_{1},\ldots{,a}_{5} \right)=(1,6,15,21,50)$ years, the per capita rate of aging is calculated as:

$k_{i}(t)=\upsilon*exp(-\upsilon*a_{i})$ for all $t$*.*

Individuals in the age group ≥50 do not move from that age group due to aging.

***Population under age-specific mortality rates***

Predictions of pneumococcal prevalence and invasive disease were done under an alternative assumption of a growing population, based the crude birth rate (8.78E-05 per day) and age-specific death rates (Table A.5) in the KHDSS population. This section summarises how the per capita rate of aging was derived under this model.

Denote the age-specific per capita mortality rate as $m(a)$ and the number of individuals of age $a$ at calendar time $t$ by $(a,t)$ . The time evolution of $N(a,t)$ is defined through the following equation:

$$\frac{dN(a,t)}{dt}=-N\left( a,t \right)m\left( a \right) (1)$$

With boundary conditions (i) $N\left( 0,t \right)=N(t)\beta$ and (ii) $N\left( a,0 \right)=N\left( 0 \right)g(a)$, where $\beta$ is the per capita rate of birth, $N(t)$ is the total population at time $t$ and $g(a)$ is the probability density of age at time 0 estimated from the KHDSS data as an exponential distribution so that $g\left( a \right)=\upsilon*exp(-\upsilon*a)$.

The rate of individuals of age $a_{i}$ (in the $i^{th}$ age group) moving to the next age group at time $t$ is given by the solution to equation (1) and its boundary conditions. For the solution, we write $M\left( a_{i} \right)=exp\left( -\int_{0}^{a_{i}} m\left( u \right)du \right)$ for survival up to age $a_{i}$, $N\left( t-a_{i} \right)\beta$ for the number of individuals (per time unit) born at time $t-a_{i}$ (if $t>a_{i}$), and $N\left( 0 \right)g\left( a_{i}-t \right)$ for the number of individuals (per time unit) of age $a_{i}-t$ (if $t\leq a_{i}$). The former deals with individuals born after the start of the simulation while the latter deals with individuals who belonged to the initial population at time $t=0$. The following numbers of individuals of age $a_{i}$ moving to the next age group at time $t$are obtained:

$$N\left( t-a_{i} \right)\beta M\left( a_{i} \right), t>a_{i},$$

$$N\left( 0 \right)g\left( a_{i}-t \right)\frac{M\left( a_{i} \right)}{M\left( a_{i}-t \right)}, t\leq a_{i}$$

The division of the second expression by $M\left( a_{i}-t \right)$ corresponds to the survival probability being conditioned on the individual(s) being alive at age $a_{i}-t$.

The total numbers of individuals moved to the next age group were further divided according to the proportions of the 14 epidemiological states in the transmission model at time $t$ to move an appropriate number of individuals to the next age group at each of the states.

In the discretized model, the per capita rate of aging is calculated as:

$k_{i}\left( t \right)=\left\{ \begin{aligned} \frac{N\left( t-a_{i} \right)\beta M\left( a_{i} \right)}{w_{i}(t)} if t>a_{i} \\ N\left( 0 \right)g\left( a_{i}-t \right)\frac{M\left( a_{i} \right)}{M\left( a_{i}-t \right)w_{i}(t)} if t\leq a_{i} \end{aligned} \right.$,

where $w_{i}(t)$ is the number of individuals in age group $i$ at time $t$.

Table A.5: Age specific death rates (per capita per day) in the KHDSS population. These are the rates used for $m\left( a \right)$ in the above equations.

| Age group | <1 | 1-5 | 6-14 | 15-20 | 21-49 | ≥50 |
| --- | --- | --- | --- | --- | --- | --- |
| Death rate | 7.18E-05 | 6.55E-06 | 2.73E-06 | 3.51E-06 | 1.21E-05 | 7.31E-05 |

**Chapter 7. Sensitivity analysis**

***Simulation under a growing population with a catch-up dose in <5 year olds***

Table A.6: Prediction of carriage prevalence before and 10-years post vaccination by age group. The table presents the predicted mean levels and 95% predictive intervals. A growing population and vaccine introduction with a catch-up dose were assumed.

|  | Pre-vaccination | | | |  | 10 years post-vaccination | | | |
| --- | --- | --- | --- | --- | --- | --- | --- | --- | --- |
| Age group | **Carriage**  **prevalence** | **VT** | **Strong NVT** | **Weak NVT** |  | **Carriage**  **prevalence** | **VT** | **Strong NVT** | **Weak NVT** |
|  |  |  |  |  |  |  |  |  |  |
| <1 | 80.8 (67.8-90.1) | 32.6 (25.1-40.5) | 37.5 (30.6-45.8) | 9.9 (7.1-13.2) |  | 82.9 (71.1-91.5) | 10.5 (2.2-21.0) | 53.6 (44.1-65.1) | 17.6 (12.1-25.6) |
| 1-5 | 72.5 (65.2-78.5) | 29.5 (23.9-35.2) | 29.7 (24.6-35.2) | 13.1 (9.6-17.0) |  | 74.6 (67.5-80.5) | 9.7 (2.1-19.2) | 42.1 (34.6-51.6) | 21.8 (16.1-28.7) |
| 6-14 | 54.0 (43.1-64.8) | 17 (13.9-21.2) | 26.8 (18.9-35.0) | 9.9 (7.4-13.5) |  | 45.9 (34.0-57.7) | 4.4 (0.9-9.1) | 28.8 (20.3-38.3) | 12.2 (9.1-16.4) |
| 15-20 | 27.9 (17.0-41.7) | 9.1 (5.7-13.9) | 13.4 (7.6-20.7) | 5.3 (3.1-8.6) |  | 18.4 (10.9-29.1) | 2.0 (0.4-4.8) | 11.2 (6.5-18.2) | 5.1 (3.0-8.3) |
| 21-49 | 25.5 (17.0-35.5) | 8.6 (5.8-12.1) | 12.0 (7.6-17.5) | 4.8 (3.0-7.2) |  | 16.9 (10.6-25.9) | 2.0 (0.4-4.4) | 10.0 (6.3-15.9) | 4.7 (2.8-7.2) |
| ≥50 | 21.0 (14.0-30.0) | 7.1 (4.7-10.1) | 9.8 (6.2-14.6) | 4.0 (2.6-6.0) |  | 17.4 (11.5-25.3) | 2.0 (0.4-4.2) | 10.4 (6.8-15.6) | 4.8 (3.1-7.3) |
|  |  |  |  |  |  |  |  |  |  |
| Overall | **44.4 (40.2-48.9)** | **15.9 (13.3-18.7)** | **20.4 (16.8-24.2)** | **44.4 (40.2-48.9)** |  | **34.9 (29.9-40.2)** | **4.0 (0.8-8.0)** | **21.1 (17.2-25.6)** | **9.6 (7.3-12.2)** |

Table A.7: Predictions of invasive pneumococcal disease (IPD) incidence before and 10-years post vaccination by age group. The table presents the predicted mean levels and 95% predictive intervals (PI). A growing population and vaccine introduction with a catch-up dose were assumed.

|  | Pre-vaccination IPD incidence | | | 10 years post-vaccination IPD incidence (95% PI) | | |  |
| --- | --- | --- | --- | --- | --- | --- | --- |
| Age groups | **VT** | **Strong NVT** | **Weak NVT** | **VT** | **Strong NVT** | **Weak NVT** | **IRR** |
|  |  |  |  |  |  |  |  |
| <1 | 67.0 | 21.0 | 7.1 | 8.6 (1.8, 18.3) | 41.1 (32.2, 55.0) | 14 (10.7, 19.0) | 0.7 (0.6, 0.8) |
| 1-5 | 39.3 | 5.1 | 0.7 | 8.1 (1.6, 17.4) | 8.8 (7.2, 11.2) | 1.3 (1.0, 1.7) | 0.4 (0.3, 0.6) |
| 6-14 | 7.3 | 1.0 | 0.0 | 1.5 (0.3, 3.1) | 1.1 (0.9, 1.3) | 0.0 (0.0, 0.0) | 0.3 (0.2, 0.5) |
| 15-20 | 1.0 | 0.0 | 0.0 | 0.2 (0.0, 0.4) | 0.0 (0.0, 0.0) | 0.0 (0.0, 0.0) | 0.2 (0.0, 0.4) |
| 21-49 | 4.2 | 0.9 | 0.0 | 0.9 (0.2, 1.7) | 0.8 (0.7, 0.9) | 0.0 (0.0, 0.0) | 0.3 (0.2, 0.5) |
| ≥50 | 9.2 | 2.8 | 4.1 | 2.5 (0.5, 4.7) | 3.0 (2.7, 3.5) | 4.9 (4.2, 6.0) | 0.7 (0.5, 0.8) |
|  |  |  |  |  |  |  |  |
| Overall | **14.8** | **2.7** | **0.9** | **2.4 (0.5, 4.9)** | **3.3 (2.8, 4.2)** | **1.1 (0.9, 1.4)** | **0.4 (0.3, 0.5)** |

**Chapter 8. Pre-vaccination IPD Incidence**

**Table A.8:** Pre-vaccination incidence of invasive pneumococcal disease (IPD) and case-to-carrier ratios by serotype group and age group. The IPD cases are from the KHDSS hospital surveillance data.

|  | Cases | | | IPD incidence*  (per 100,000 per year) | | | Case-to-carrier ratio**  (per 10,000) | | |
| --- | --- | --- | --- | --- | --- | --- | --- | --- | --- |
| Age group  (years) | **VT** | **Strong**  **NVT** | **Weak**  **NVT** | **VT** | **Strong**  **NVT** | **Weak**  **NVT** | **VT** | **Strong**  **NVT** | **Weak**  **NVT** |
| <1 | 19 | 6 | 2 | 67.0 | 21.0 | 7.1 | 4.28 (3.03-6.16) | 1.02 (0.73 -1.40) | 0.90 (0.65-1.31) |
| 1-5 | 54 | 7 | 1 | 39.3 | 5.1 | 0.7 | 1.44 (1.11-1.91) | 0.15 (0.12-0.20) | 0.06 (0.04-0.09) |
| 6-14 | 15 | 2 | 0 | 7.3 | 1.0 | 0.0 | 0.32 (0.24-0.40) | 0.02 (0.02-0.04) | 0.00 (0.00-0.00) |
| 15-20 | 1 | 0 | 0 | 1.0 | 0.0 | 0.0 | 0.09 (0.06-0.15) | 0.00 (0.00-0.00) | 0.00 (0.00-0.00) |
| 21-49 | 9 | 2 | 0 | 4.2 | 0.9 | 0.0 | 0.40 (0.27-0.61) | 0.06 (0.04-0.10) | 0.00 (0.00-0.00) |
| ≥50 | 7 | 2 | 3 | 9.2 | 2.8 | 4.1 | 1.07 (0.73-1.65) | 0.23 (0.15-0.38) | 0.87 (0.57-1.37) |

*(*) Average across 3 years (2008,2009,2010) to represent pre-vaccination IPD incidence.*

*(**) These are estimated based on modelled carriage incidence and the observed IPD incidence. Figures in the brackets represent the 95% credible intervals.*
